# High-Sensitivity Real-Time Tracking System for High-Speed Pipeline Inspection Gauge

**DOI:** 10.3390/s19030731

**Published:** 2019-02-11

**Authors:** Guanyu Piao, Jingbo Guo, Tiehua Hu, Yiming Deng

**Affiliations:** 1 Department of Electrical Engineering, Tsinghua University, Beijing 100084, China; pgy14@mails.tsinghua.edu.cn (G.P.); huthjk@sina.com (T.H.); 2 Department of Electrical and Computer Engineering, Michigan State University, East Lansing, MI 48824, USA; dengyimi@egr.msu.edu

**Keywords:** high-sensitivity detection, above ground marker, pipeline inspection gauge, data fusion model, search coil sensor, ELF tracking

## Abstract

Real-time tracking of pipeline inspection gauges (PIGs) is an important aspect of ensuring the safety of oil and gas pipeline inline inspections (ILIs). Transmitting and receiving extremely low frequency (ELF) magnetic signals is one of the preferred methods of tracking. Due to the increase in physical parameters of the pipeline including transportation speed, wall thickness and burial depth, the ELF magnetic signals received are short transient (1-second duration) and very weak (10 pT), making the existing above-ground-marker (AGM) systems difficult to operate correctly. Based on the short transient very weak characteristics of ELF signals studied with a 2-D finite-element method (FEM) simulation, a data fusion model was derived to fuse the envelope decay rates of ELF signals by a least square (LS) criterion. Then, a fast-decision-tree (FDT) method is proposed to estimate the fused envelope decay rate to output the maximized orthogonal signal power for the signal detection through a determined topology and a fast calculation process, which was demonstrated to have excellent real-time detection performance. We show that simulation and experimental results validated the effectiveness of the proposed FDT method, and describe the high-sensitivity detection and real-time implementation of a high-speed PIG tracking system, including a transmitter, a receiver, and a pair of orthogonal search coil sensors.

## 1. Introduction

Today, over 70 percent of oil and gas transportation relies on a total of over 3,500,000 km of pipelines in 120 countries, which is the safest, most reliable and efficient method, compared with highways, railways and marine transport, and so on [1]. To meet the rapidly growing demand for oil and gas, the construction of trunk pipelines which can operate in high-pressure, high-speed and high-capacity conditions has been increasing in recent years, giving the pipelines larger diameters, thicker pipe walls, deeper burial depths and larger transportation distances [2,3,4,5]. Due to corrosion and high-pressure loadings on the pipe wall, pipeline failures including defects, deformations, blockages, leakages and explosions occur every year, resulting in serious risks to pipeline safety [6,7,8]. In China, it was reported that there was a pipeline failure every 4 km [9], and in the USA, the economic and human losses due to the significant pipeline incidents in the past 20-year period are $7 billion and 330 fatalities along with 1292 injuries, respectively [10]. Pipeline inspection gauges (PIGs) are periodically used for pipeline in-line inspections (ILIs) to minimize these risks and maintain pipeline integrity [11,12,13,14,15,16]. However, during the pipeline ILI operation, the PIG is likely to be blocked by the deformed pipelines, while an odometer installed in the PIG can only record the PIG position offline, because the shielding effect of the steel pipe wall makes it difficult to transmit the position information from inside the pipeline to the ground through wireless communication technologies, such as Wi-Fi, Bluetooth, or 3G and 4G networks [17,18]. Thus, the pipeline ILI usually needs the use of above-ground-marker (AGM) technologies to track the position of the PIG in real time [19,20].

**Figure 1:**
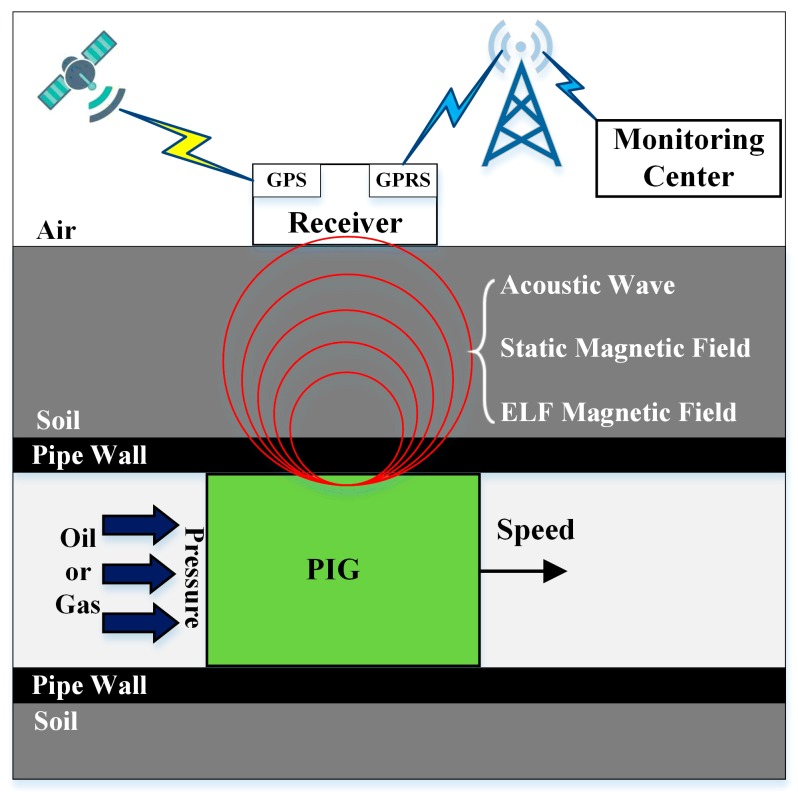
Working principle of above-ground-marker (AGM) system.

Existing AGM methods utilize acoustic wave, static magnetic field, extremely low frequency (ELF) magnetic fields for PIG localization and tracking, and have been widely practiced in the oil and gas industry [21,22,23,24,25,26,27]. Here, the working principle of the AGM system is shown in Figure 1. The PIG is propelled by the flowing medium and the speed is mainly determined by the pressure inside the pipeline. A receiver placed above the ground detects the tracking signals generated by the PIG moving right below, and records the PIG position simultaneously. With the help of the global position system (GPS) and general packet radio service (GPRS) technologies, all receivers along the pipeline usually with an interval of 1 km can send the current location and local time of the PIG to the monitoring center to form a tracking network, and the information will also be used to compensate for the accumulated locating error caused by the PIG odometer.

The acoustic wave method is used to detect the acoustic wave generated by the friction between the PIG and the pipe wall. In [21,22], it is used to improve the tracking precision of the underground PIG, and in [23], a micro-electro-mechanical-system (MEMS) acoustic vector sensor is found to have advantages of high sensitivity for detecting low-frequency acoustic signals. However, the acoustic signals generated by the moving PIG are easily cluttered by the environmental acoustic sources, such as moving vehicles, trains and airplanes, and it produces a high number of false alarms. The static magnetic field method is used to detect the static magnetic field generated by the permanent magnets assembled in the PIG. In [24,25], experimental results showed that this method was effective when the magnetic signals emitted by the permanent magnets were strong enough. Furthermore, it is only applicable to certain types of PIG due to the requirement to carry heavy and bulky permanent magnets, thus limiting the scope of application. To overcome the aforementioned challenges, the ELF method is introduced, which is a single-frequency magnetic signal transmission and reception method that is suitable for most types of PIG [26,27]. A small and lightweight transmitter equipped in the PIG generates the ELF magnetic field and the receiver detects the ELF magnetic field using magnetic sensors and real-time signal detection methods. Comparing to high-frequency magnetic field, the ELF magnetic field with frequencies between 22 Hz and 24 Hz has a good penetrating ability for signal transmitting through steel pipe wall and soil, and is less susceptible to environmental interferences [28,29]. In [30], the tracking and localization of the in-pipe PIG is realized by applying the equivalent magnetic dipole model and the inverse calculation of the ELF magnetic field. In [27,28,30], experimental results showed that the existing ELF tracking systems worked well for long-duration and high-magnitude ELF magnetic signals. However, due to the higher speed of the PIG during the pipeline ILI operation, the received time-domain ELF signals become a transient signal with a very short duration [29]. Due to the thicker pipe wall and deeper burial depth of pipelines, as well as the desired low power consumption requirement of the transmitter for the long working time, the magnitude of the received ELF magnetic field is very low and immersed in strong background noise, leading to a very poor signal-to-noise ratio (SNR) [31]. The increases of the speed, pipe wall thickness, and burial depth means the existing ELF tracking systems still face the challenges of detecting the short transient very weak ELF magnetic signals.

According to the signal detection theory, solving these challenges requires the use of high-sensitivity magnetic sensors with advanced signal detection methods, so that the probability of detection (POD) of the short transient very weak ELF signals can be high enough for a given low probability of false alarm (PFA). There are several high-sensitivity magnetic sensors that can be used to detect very weak magnetic signals, such as the search coil sensor, fluxgate magnetometer and SQUID sensor [32,33]. A search coil sensor can detect magnetic fields as weak as 10^−2^ pT with a frequency range from 1 Hz to 1 MHz. A fluxgate magnetometer can detect magnetic fields from 10 to 10^9^ pT, while the frequency response is limited by the excitation field and response time of ferromagnetic materials. A SQUID sensor is more sensitive at low frequencies (<1 Hz) and the sensitivity is as low as 10^−2^ pT. However, the need for liquid-helium coolant makes the SQUID sensor heavy and inconvenient. As for the signal detection, the generalized likelihood ratio (GLR) is considered to be one of the typical signal detection methods [34,35]. Poor et al. [36] develops the quickest change detection method based on the distribution of test statistics. Wavelet transform becomes an effective tool for joint time-frequency analysis and has a broader application in the diagnosis of mechanical faults and the evaluation of non-destructive testing (NDT) signals [37,38,39]. Marius Birsan et al. [40] presents an underwater detection of ELF electromagnetic field signals from ships using the cross wavelet transform and wavelet coherence method for high SNR situations. Qin et al. [41] present transient weak fault feature extraction based on an optimized Morlet wavelet transform and kurtosis index. Meanwhile, a maximum likelihood (ML) estimator is also developed to extract the amplitude from fault-generated transient impulses in [42].

This paper proposes a high-sensitivity real-time tracking system for high-speed PIG to detect short transient very weak ELF magnetic signals. A 2-D finite-element method (FEM) simulation was conducted to understand the relationships between the time-domain ELF signals and the physical parameters of the pipeline, including transportation speed, wall thickness, and burial depth. According to the short transient very weak characteristics of ELF signals, a data fusion model was derived to well represent the ELF signals by fusing the envelope decay rates through a least square (LS) criterion. Then, a fast-decision-tree (FDT) method is proposed to detect the ELF signals. The main differences of the FDT method are: (1) it first estimates the fused envelope decay rate to maximize the orthogonal signal power of ELF signals, and the maximized orthogonal signal power is used as the test statistic for the signal detection, which provides an excellent detection performance; and (2) the deterministic topology and fast calculation process of the FDT method avoid massive matrix inversion and multiplication, which significantly reduces the computation costs and provides excellent real-time performance. The detection performance of the proposed FDT method is discussed, with a comparison between other existing detection methods as well as theoretical bounds through Monto-Carlo simulations. In consideration of high sensitivity as well as convenience, a pair of orthogonal search coil sensors was designed to receive the very weak ELF magnetic signals. Then, an ELF tracking system including an ELF transmitter, an ELF receiver and a pair of orthogonal search coil sensors was developed and tested, and the experimental results validate the effectiveness of the proposed FDT method. The main reason for the excellent detection performance obtained is discussed through the analyses of the normalized power spectrum, estimation performance of speed and SNR. Finally, the high-sensitivity detection and real-time implementation of high-speed PIG tracking system is achieved.

## 2. Simulation and Signal models

### 2.1. 2-D FEM Simulation Studies

**Figure 2:**
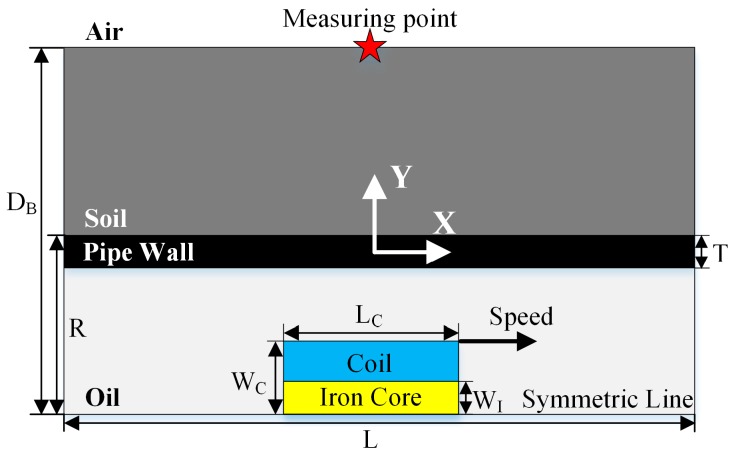
Schematic of 2-D finite-element method (FEM) simulation model.

In order to study the characteristics of the ELF signals for further developing a targeted signal detection method as well as sensing system, simulation studies of 2-D harmonic magnetic analyses are conducted using ANSYS software, and the 2-D FEM model is shown in Figure 2. The FEM model is axisymmetric and consists of transmitter, oil, steel pipe wall, soil and air. The cylindrical transmitter supported by the PIG’s mechanical connecting structure is located at the symmetric line of the pipeline. The transmitter consists of a transmitting coil and an iron core which has a high permeability to increase the strength of the ELF magnetic field. The transmitting coil is fed a sinusoidal 4 mA (root mean square value) current with frequency of 23 Hz. The simulated speed of the transmitter ranges from 1 m/s to 15 m/s, and the ELF signals in the pipe longitudinal direction (*X*-axis direction) and pipe radial direction (*Y*-axis direction) are calculated at the point above the soil surface (location highlighted by “star” in Figure 2). The element and boundary condition of the FEM simulation is set to PLANE53 and flux-parallel condition, respectively. The material parameters including conductivity, permeability and mesh size are listed in Table 1, and the model geometry is listed in Table 2.

**Figure 3:**
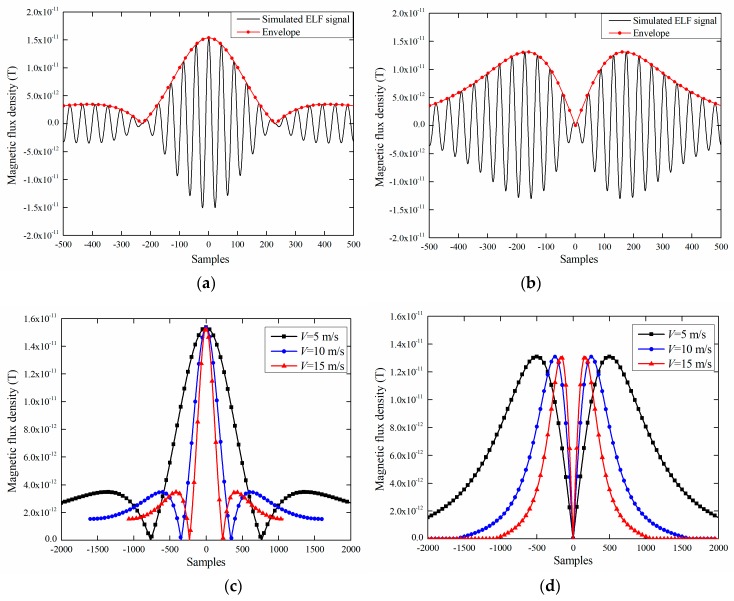
Simulated time-domain extremely low frequency (ELF) signals and envelopes of *X*-axis and *Y*-axis, respectively: (**a**) *X*-axis signals in 15 m/s; (**b**) *Y*-axis signals in 15 m/s; (**c**) *X*-axis envelopes in 5 m/s, 10 m/s and 15 m/s. (**d**) *Y*-axis envelopes in 5 m/s, 10 m/s and 15 m/s.

The simulated ELF signals of the *X*-axis and *Y*-axis, when the speed is 15 m/s, pipe wall thickness is 15 mm and burial depth is 5 m, are shown in Figure 3a,b. The envelopes of the *X*-axis and *Y*-axis ELF signals with three different speeds are shown in Figure 3c,d. The sampling interval of the 2-D FEM simulation is set to 1 millisecond, and a sample equal to 0 means that the ELF transmitter passes right below the measuring point. Figure 3a,b shows that the number of samples of the ELF signals is 1000 points, corresponding to a signal duration as low as 1 second, and the amplitudes are as low as 15 pT. Figure 3c,d shows that the steepness of the envelopes is closely related to the speed of the transmitter, and the envelopes appear to be short transient as the transmitter moves faster.

**Figure 4:**
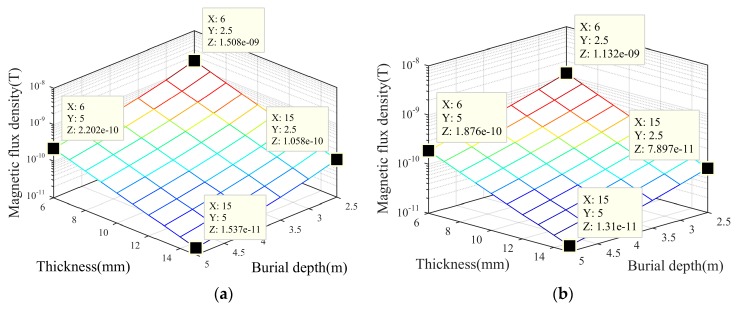
Amplitudes of *X*-axis and *Y*-axis ELF signals change with pipe wall thickness and burial depth, respectively: (**a**) *X*-axis; (**b**) *Y*-axis.

The amplitudes of the ELF signals of the *X*-axis and *Y*-axis, changing with the pipe wall thickness and burial depth, are shown in Figure 4a,b. The coordinate of thickness and burial depth is set to a linear scale, while that of the magnetic flux density is set to a logarithmic scale, and the amplitudes decrease logarithmically as the thickness and burial depth increase linearly. Taking the *X*-axis results as an example, compared to the amplitude for thin wall thickness (6 mm) and shallow burial depth (2.5 m) which is about 1000 pT, the amplitudes for thick wall thickness (15 mm) or deep burial depth (5 m) are about 100 pT, which decreases by an order of magnitude. Then, the amplitude for thick wall thickness and deep burial depth is about 10 pT, which decreases by two orders of magnitude. The trend of *Y*-axis results is the same as for the *X*-axis. The simulation results discussed above show that under the circumstances of a high speed of 15 m/s, the conditions of a thicker pipe wall and deeper burial depth will cause the ELF signals to become short transient in the envelope and very weak in amplitude.

### 2.2. Data Fusion Model

The very weak ELF signals are easily interfered with by strong narrow-band background noise. The ELF signals of *X*-axis and *Y*-axis with noise can be expressed as:

(1) $$x[n]=s_{x}[n]+w_{x}[n]\;\;n=-\frac{N_{x}}{2},\ldots ,-1,\;0,\;1,\ldots ,\frac{N_{x}}{2}$$

(2) $$y[n]=s_{y}[n]+w_{y}[n]\;n=-\frac{N_{y}}{2},\ldots ,-1,\;0,\;1,\ldots ,\frac{N_{y}}{2}$$

where *w_x_*[*n*] and *w_y_*[*n*] are narrow-band background noise, *N_x_* and *N_y_* are the number of samples along *X*-axis and *Y*-axis, respectively. Table 3 summarizes the important notations and their definitions used in this article.

As shown in Figure 3a,b, the time-domain ELF signals have two main properties including envelope and oscillation. Here, a Morlet mother wavelet is used to represent the steepness of the envelope as well as the oscillation:

(3) $$\psi (t)=e^{-\beta t^{2}/2}\cos\omega t$$

where *β* is defined as the envelope decay rate, and the exponential component is used to represent the steepness, while the cosine component is used to represent the single-frequency oscillation. There are a main lobe peak and two side lobe peaks in the *X*-axis signals. Since the two side lobe peaks are much smaller than the main lobe peak and are more susceptible to noise interference, we set up the *X*-axis signal model with the main lobe peak:

(4) $$s_{x}[n]=A_{x}e^{-\beta_{x}{\left[n/f_{s}\right]}^{2}}\cos[\omega_{0}n/f_{s}+\varphi_{0}]$$

taking the origin and two peak points as cut-off points, the *Y*-axis signal model is set by four intervals:

(5) $$s_{y}[n_{1},n_{2}]=\{\begin{array}{l} A_{y}e^{-\;\beta_{yl}{\left[n_{1}/f_{s}\right]}^{2}}\cos[\omega_{0}n_{2}/f_{s}+\varphi_{0}]\;\;\;\;n_{1}=-\frac{N_{y}-M_{y}}{2},\cdots ,-1,0\;\;\;n_{2}=-\frac{N_{y}}{2},\cdots ,-\frac{M_{y}}{2}\\ A_{y}e^{-\;\beta_{yh}{\left[n_{1}/f_{s}\right]}^{2}}\cos[\omega_{0}n_{2}/f_{s}+\varphi_{0}]\;\;\;\;n_{1}=0,1,\cdots ,\frac{M_{y}}{2}\;\;\;n_{2}=-\frac{M_{y}}{2},\cdots ,-1,0\\ A_{y}e^{-\;\beta_{yh}{\left[n_{1}/f_{s}\right]}^{2}}\cos[\omega_{0}n_{2}/f_{s}+\varphi_{0}]\;\;\;\;n_{1}=-\frac{M_{y}}{2},\cdots ,-1,0\;\;\;n_{2}=0,1,\cdots ,\frac{M_{y}}{2}\\ A_{y}e^{-\;\beta_{yl}{\left[n_{1}/f_{s}\right]}^{2}}cos[\omega_{0}n_{2}/f_{s}+\varphi_{0}]\;\;\;\;n_{1}=0,1,\cdots ,\frac{N_{y}-M_{y}}{2}\;\;\;n_{2}=\frac{M_{y}}{2},\ldots ,\frac{N_{y}}{2} \end{array}$$

where *n*_1_ is window variable of the exponential function in *Y*-axis signal model, *n* and *n*_2_ are both window variables of the sine function in *X*-axis and *Y*-axis signal models, respectively. The normalized angular frequency is *ω*_0_ = 2*πf*_0_/*f*_s_ in unit of rad/sample. The ELF signal frequency *f*_0_ is 23 Hz and the sampling frequency *f*_s_ is 1 kHz. *A_x_* and *A_y_* are amplitudes of the *X*-axis and *Y*-axis signals, respectively, and variable *M_y_* is the coordinate intervals of the two peaks in the *Y*-axis. Variable *β_x_* is the envelope decay rate of main lobe peak of *X*-axis, and *β_yl_* and *β_yh_* are used to represent the *Y*-axis envelopes deviating from the origin and facing the origin, respectively.

It is noted that the envelope decay rates (*β_x_*, *β_yl_* and *β_yh_*) increase as the speed of the transmitter increases, as shown in Figure 3c,d. In order to simplify these three parameters to match the speed of the transmitter well, the fused envelope decay rate is derived here by a LS criterion. Taking the *X*-axis signal as an example, the LS criterion minimizes error function by estimating the parameters (*A_x_*, *φ*_0_ and *β_x_*) in *s_x_*[*n*]:

(6) $$J_{x}=\sum_{n=-\frac{N_{x}}{2}}^{\frac{N_{x}}{2}}{\left(s_{x}[n]-x[n]\right)}^{2}.$$

Then, Equation (6) can be decomposed into the linear form as:

(7) $$s_{x}[n]=H_{x}\alpha_{x}$$

where the observation matrix is:

(8) $$H_{x}=[H_{x1},H_{x2}]=[\begin{array}{ll} e^{-\beta_{x}{\left[-\frac{N_{x}}{2f_{s}}\right]}^{2}}\cos[\omega_{0}(-\frac{N_{x}}{2})] & \;\;e^{-\beta_{x}{\left[-\frac{N_{x}}{2f_{s}}\right]}^{2}}\sin[\omega_{0}(-\frac{N_{x}}{2})]\;\;\\ \;\;\;\;\;\;\;\;\;\;\;\;\;\;\;\;\;\;\;\;\;\;\;\;\;\;\vdots & \;\;\;\;\;\;\;\;\;\;\;\;\;\;\;\;\;\;\;\;\;\;\;\;\;\;\vdots \\ e^{-\beta_{x}{\left[\frac{1}{f_{s}}(-1)\right]}^{2}}\cos[\omega_{0}\times (-1)] & \;\;\;\;\;e^{-\beta_{x}{\left[\frac{1}{f_{s}}(-1)\right]}^{2}}\sin[\omega_{0}\times (-1)]\\ e^{-\beta_{x}{\left[\frac{1}{f_{s}}\times 0\right]}^{2}}\cos[\omega_{0}\times 0] & \;\;\;e^{-\beta_{x}{\left[\frac{1}{f_{s}}\times 0\right]}^{2}}\sin[\omega_{0}\times 0]\\ e^{-\beta_{x}{\left[\frac{1}{f_{s}}(1)\right]}^{2}}\cos[\omega_{0}\times 1] & \;\;\;e^{-\beta_{x}{\left[\frac{1}{f_{s}}(1)\right]}^{2}}\sin[\omega_{0}\times 1]\\ \;\;\;\;\;\;\;\;\;\;\;\;\;\;\;\;\;\;\;\;\;\;\;\;\;\;\vdots & \;\;\;\;\;\;\;\;\;\;\;\;\;\;\;\;\;\;\;\;\;\;\;\;\;\;\vdots \\ e^{-\beta_{x}{\left[\frac{N_{x}}{2f_{s}}\right]}^{2}}\cos[\omega_{0}(\frac{N_{x}}{2})] & \;\;\;e^{-\beta_{x}{\left[\frac{N_{x}}{2f_{s}}\right]}^{2}}\sin[\omega_{0}(\frac{N_{x}}{2})] \end{array}].$$

Thus, the estimation of **α_x_** (*A_x_* and *φ*_0_) is expressed as:

(9) $${\hat{\alpha }}_{x}=(H_{x}{}^{T}H_{x}{)}^{-1}H_{x}{}^{T}x.$$

Calculating the minimization of (6) is equal to calculate the maximum estimation of the *X*-axis signal energy, which is derived as:

(10) $${\hat{P}}_{x}=x^{T}H_{x}{\left(H_{x}{}^{T}H_{x}\right)}^{-1}H_{x}{}^{T}x.$$

Thus, the estimation of *β_x_* can be estimated by maximizing (10) as:

(11) $${\hat{\beta }}_{x}=\arg\;\underset{\beta_{x}}{\max}[{\hat{P}}_{x}=x^{T}H_{x}{\left(H_{x}{}^{T}H_{x}\right)}^{-1}H_{x}{}^{T})x].$$

Similarly, the estimation of *β_yl_* and *β_yh_* are expressed as:

(12) $$\left({\hat{\beta }}_{yl},{\hat{\beta }}_{yh})=\arg\;\underset{\left(\beta_{yl},\beta_{yh}\right)}{\max}[{\hat{P}}_{y}=y^{T}H_{y}{\left(H_{y}{}^{T}H_{y}\right)}^{-1}H_{y}{}^{T})y\right]$$

where **H_y_** is transformed by *s_y_*[*n*_1_,*n*_2_] and the transformation process is similar to **H_x_**.

The purpose of the fusion is to find a relationship between these three envelope decay rates to minimize the error functions of the *X*-axis and *Y*-axis simultaneously. Assume that *β_x_* = *k_xy_β_yh_* = *k_y_β_yl_*, and define the estimation of the orthogonal signal power as:

(13) $${\hat{P}}_{xy}=\frac{{\hat{P}}_{x}}{N_{x}}+\frac{{\hat{P}}_{y}}{N_{y}}.$$

**Figure 5:**
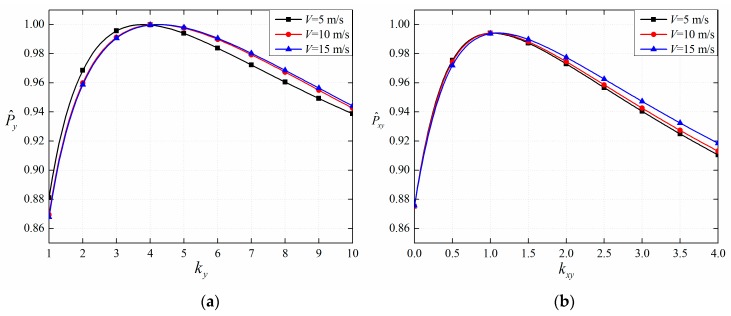
The relationships between *k_y_*, *k_xy_* and normalized ${\hat{P}}_{y}$, ${\hat{P}}_{\mathrm{xy}}$: (**a**) *k_y_*; (**b**) *k_xy_* and ${\hat{P}}_{\mathrm{xy}}$.

Then, the relationship between *k_y_* and normalized ${\hat{P}}_{y}$ for three different speeds is shown in Figure 5a, and the relationship between *k_xy_* and normalized ${\hat{P}}_{\mathrm{xy}}$ for three different speeds is shown in Figure 5b.

Figure 5a shows that ${\hat{P}}_{y}$ reaches a maximum when *k_y_* is around 4 in the cases of 5 m/s, 10 m/s and 15 m/s. Figure 5b shows that ${\hat{P}}_{\mathrm{xy}}$ reaches a maximum when *k_xy_* is around 1 in the cases of 5 m/s, 10 m/s and 15 m/s. Thus, the fused envelope decay rate *β_xy_* is defined through the linear relationship as:

(14) $$\beta_{xy}=\beta_{x}=\beta_{yh}=4\beta_{yl}.$$

The linear relationship has two main meanings: (1) the three envelope decay rates of the derived data fusion model have the similar varying trend as the speed of the transmitter changes, and the varying trend can be represented by a fixed linear relationship; and (2) the linear relationship satisfies the LS criterion by maximizing ${\hat{P}}_{x}$, ${\hat{P}}_{y}$ and ${\hat{P}}_{\mathrm{xy}}$, which contributes to the optimal estimation of the fused envelope decay rate.

## 3. Fast decision Tree Method

The data fusion model sets up the relationship between the orthogonal ELF signals and the fused envelope decay rate. According to the Equations from (10) to (14), maximizing the orthogonal signal power is equivalent to estimating the fused envelope decay rate, which also improves the ability of extracting the ELF signals from strong narrow-band background noise. Thus, the optimal estimation of the fused envelope decay rate is critical, and the maximized orthogonal signal power can be used as the test statistic for the signal detection, which is also the detection principle of the proposed FDT method. First, the fast calculation process of the proposed FDT method is derived as follows. Take the *X*-axis ELF signal as an example, according to the Equation (8), we have:

(15) $$\begin{array}{l} H_{x1}{}^{T}H_{x2}=H_{x2}{}^{T}H_{x1}=\sum_{n=-\frac{N_{x}}{2}}^{\frac{N_{x}}{2}}e^{-\beta_{x}{\left[n/f_{s}\right]}^{2}}\cos[\omega_{0}n/f_{s}]\times e^{-\beta_{x}{\left[n/f_{s}\right]}^{2}}\sin[\omega_{0}n/f_{s}]\\ \;\;\;\;\;\;\;\;\;\;\;\;\;\;\;\;\;\;\;\;\;\;\;\;\;\;\;\;\;\;\;=\;\sum_{n=-\frac{N_{x}}{2}}^{\frac{N_{x}}{2}}e^{-2\beta_{x}{\left[n/f_{s}\right]}^{2}}\sin[2\omega_{0}n/f_{s}]\approx 0 \end{array}$$

Thus, simplify the Equation (10) as:

(16) $${\hat{P}}_{x}=x^{T}H_{x}{\left(H_{x}{}^{T}H_{x}\right)}^{-1}H_{x}{}^{T}x\approx x^{T}H_{x}{\left[\begin{array}{l} H_{x1}{}^{T}H_{x1}\;\;\;\;\;\;0\\ \;\;\;0\;\;\;\;\;\;\;H_{x2}{}^{T}H_{x2} \end{array}\right]}^{-1}H_{x}{}^{T}x$$

where an approximate equation can be derived as:

(17) $$P_{H_{x}}=H_{x1}{}^{T}H_{x1}\approx H_{x2}{}^{T}H_{x2}$$

where $P_{H_{x}}$ is defined as the energy of *X*-axis observation vector. Then, we have:

(18) $${\hat{P}}_{x}\approx \frac{xH_{x}{}^{T}H_{x}{}^{T}x}{P_{H_{X}}}=\frac{{\left(H_{x}{}^{T}x\right)}^{T}H_{x}{}^{T}x}{P_{H_{X}}}.$$

Similarly, the process of the *Y*-axis ELF signal is as follows:

(19) $$P_{H_{y}}=H_{y1}{}^{T}H_{y1}\approx H_{y2}{}^{T}H_{y2}.$$

(20) $${\hat{P}}_{y}\approx \frac{yH_{y}{}^{T}H_{y}{}^{T}y}{P_{H_{y}}}=\frac{{\left(H_{y}{}^{T}y\right)}^{T}H_{y}{}^{T}y}{P_{H_{y}}}.$$

**Figure 6:**
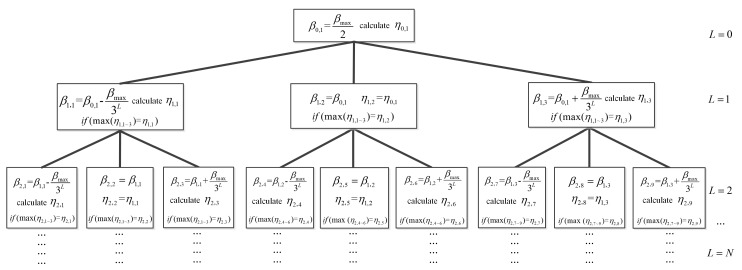
Topological structure of the fast-decision-tree (FDT) method.

Next, the topology of the FDT method is derived as follows. The FDT method evenly divides the fused envelope decay rate into three categories at each of the tree nodes, and then calculates the normalized signal energy of the three child nodes, which is defined as:

(21) $$\eta =\left(\frac{{\hat{P}}_{x}}{P_{x}}+\frac{{\hat{P}}_{y}}{P_{y}}\right)/2.$$

where *P_x_* = **x***^T^***x** and *P_y_* = **y***^T^***y** are received signal energy of *X*-axis and *Y*-axis signals, respectively. From Equations of (10) and (12), ${\hat{P}}_{x}$ and ${\hat{P}}_{y}$ are always smaller than *P_x_* and *P_y_*, respectively, and that *η* ∈ (0,1). The FDT method estimates the fused envelope decay rate through the decision rule, which is defined as determining the maximum *η* of the three child nodes as the next iteration direction. Here, the evolution of the FDT method and the decision rule are clearly shown in Figure 6. Variables *L* and *n* in *β_L,n_* and *η_L,n_* represent the tree layer and tree node. *β_max_* corresponds to the maximum speed of the transmitter. As shown in Figure 6, the FDT method sets the maximum *η* as the next iteration direction, which is equivalent to selecting and estimating the fused enveloped decay rate that satisfies the LS criterion through the Equations (11) and (12).

The iteration stopping criterion is defined as follows: the FDT method calculates the *η* of three child nodes and then distinguishes them into three values: *η_L,max_*, *η_L,med_* and *η_L,min_*. The iteration is stopped when the *η* meets:

(22) $$\Delta \eta =\eta_{L,\max}-\eta_{L,\mathrm{med}}\leq 0.001.$$

The varying trend of *η* at each FDT layer through the iteration procedure is listed in Table 4. The speed range of the FDT topology is set from 1 m/s to 15 m/s, and *β_max_* is set to 50.

Table 4 shows in detail that the FDT method estimates *β_xy_* by comparing and determining the maximum (which is bold) of the *η* in each layer. In layer 4, the iteration of the FDT meets the stopping criterion with small errors for Δ*η* and Δ*β_xy_*.

According to the topology and fast calculation process, once the maximum speed of the transmitter is confirmed, *β*_max_ of the FDT and *β_xy_* of each FDT nodes are both confirmed. Meanwhile, since the parameters including *N_x_*, *f_s_*, *ω*_0_ are priori knowledge, $P_{H_{X}}$ and $P_{H_{Y}}$ are also priori knowledge by the equation as:

(23) $$(P_{H_{x}},P_{H_{Y}})=f(\beta_{xy},N_{x},f_{s},\omega_{0}).$$

Therefore, the computation cost of the proposed FDT method is derived as:

(24) $$\left\{ \begin{array}{l} C_{m}=2N_{x}+3,\;C_{a}=2N_{x}+1\;\;\;\;\;\;\;\;\;\;\;\;\;\;\;\;\;\;\;\;\;\;\;\;\;\;\;\mathrm{to}\;\mathrm{get}\;{\hat{P}}_{x}\;\mathrm{in}\;(18)\\ C_{m}=2N_{y}+3,\;C_{a}=2N_{y}+1\;\;\;\;\;\;\;\;\;\;\;\;\;\;\;\;\;\;\;\;\;\;\;\;\;\;\;\mathrm{to}\;\mathrm{get}\;{\hat{P}}_{y}\;\mathrm{in}\;(20)\\ C_{m}=N_{x}+N_{y},\;C_{a}=N_{x}+N_{y}+2\;\;\;\;\;\;\;\;\;\;\;\;\;\;\;\;\;\;\mathrm{to}\;\mathrm{get}\;P_{x}\;\mathrm{and}\;P_{y}\;\mathrm{in}\;(21)\\ C_{m}=3N_{x}+3N_{y}+3,\;C_{a}=3N_{x}+3N_{y}+3\;\;\;\mathrm{to}\;\mathrm{get}\;\eta \;\mathrm{in}\;(21) \end{array}\right.$$

where *C_m_* and *C_a_* are computation costs of multiplication and addition, respectively. It is worth noting that the proposed FDT method only requires the computation costs of *C_m_* = 3*N_x_* + 3*N_y_* + 3 and *C_a_* = 3*N_x_* + 3*N_y_* + 3 to calculate one *η* at a time instead of massive matrix inversion and multiplication mentioned in (10) and (12), which is the key for ensuring a real-time performance.

Finally, the FDT method detects the ELF signals through a hypothesis test defined as:

(25) $$\left\{ \begin{array}{l} H_{0}:{\hat{P}}_{xy}=\frac{{\hat{P}}_{x}}{N_{x}}+\frac{{\hat{P}}_{y}}{N_{y}}<\hat{\gamma }\\ H_{1}:{\hat{P}}_{xy}=\frac{{\hat{P}}_{x}}{N_{x}}+\frac{{\hat{P}}_{y}}{N_{y}}>\hat{\gamma } \end{array}\right.$$

where $\hat{\gamma }$ is a threshold; *H*_0_ is referred as the null hypothesis which shows nonexistence of signals and *H*_1_ as the alternative hypothesis which shows existence of signals. The Newman-Pearson criterion is then used to build the threshold which maximizes POD for a given PFA, and the relationship between the threshold $\hat{\gamma }$ and PFA is defined as:

(26) $$\mathrm{PFA}=Q(\frac{\hat{\gamma }-\hat{\mu }}{\hat{\sigma }})=\int_{\frac{\hat{\gamma }-\hat{\mu }}{\hat{\sigma }}}^{\infty }\frac{1}{\sqrt{2\pi }}\exp(-\frac{1}{2}t^{2})dt$$

where $\hat{\mu }$ and $\hat{\sigma }$ are the mean and the variance of the test statistic due to narrow-band background noise, respectively, *Q*(*x*) is called the right-tail probability function. The threshold is calculated by (26) for a given PFA before the signal detection.

## 4. Performance Evaluation

### 4.1. Detection Methods and Theoretical Bounds

A maximum likelihood (ML)-based detection method sets the estimated amplitude of the ELF signals as the test statistic, which is based on the time-domain signals without considering the short transient characteristics of the envelopes. A discrete wavelet transform (DWT)-based detection method reconstructs ELF signal envelopes using the wavelet coefficient at the characteristic scales, using a Symlet wavelet based on the time-frequency analysis. Meanwhile, two theoretical bounds are used for comparison with the proposed FDT method’s results. The upper bound is derived from the matched filter (MF) method:

(27) $$D_{MF}=\frac{s_{x}x}{\sigma_{x}{}^{2}}+\frac{s_{y}y}{\sigma_{y}{}^{2}}.$$

where **s_x_** and **s_y_** are the noiseless ELF signals and regarded as a prior known here; *σ_x_* and *σ_y_* are noise variances for the *X*-axis and *Y*-axis signals, respectively. The lower bound is derived from the energy detection (ED) method:

(28) $$D_{ED}=\frac{x^{T}x}{\sigma_{x}{}^{2}}+\frac{y^{T}y}{\sigma_{y}{}^{2}}.$$

The estimation of SNR is defined as:

(29) $$\mathrm{SNR}=10\log_{10}\left(\frac{s_{x}{}^{T}s_{x}+s_{y}{}^{T}s_{y}}{w_{x}{}^{T}w_{x}+w_{y}{}^{T}w_{y}}\right)\approx 10\log_{10}\left(\frac{{\hat{P}}_{x}+{\hat{P}}_{y}}{N_{x}{\hat{\sigma }}_{x}+N_{y}{\hat{\sigma }}_{y}}\right).$$

where **w_x_** and **w_y_** are the narrow-band background noise, which have the same definition as Equations (1) and (2). The mean of the narrow-band background noise can be considered to be zero, while the variances including ${\hat{\sigma }}_{x}$ and ${\hat{\sigma }}_{y}$ are obtained from the long-time observation. It is worth noting that ${\hat{P}}_{x}$ and ${\hat{P}}_{y}$ calculated from the FDT method are close to the noiseless ELF signal energy, and the estimation performance of the SNR will be discussed in Section 5.

### 4.2. Monte Carlo Simulation Study

**Figure 7:**
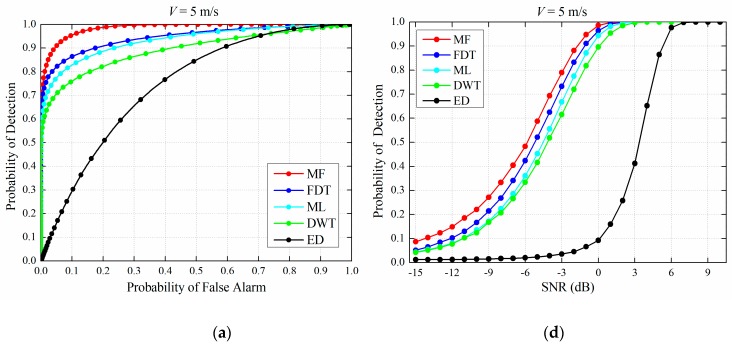

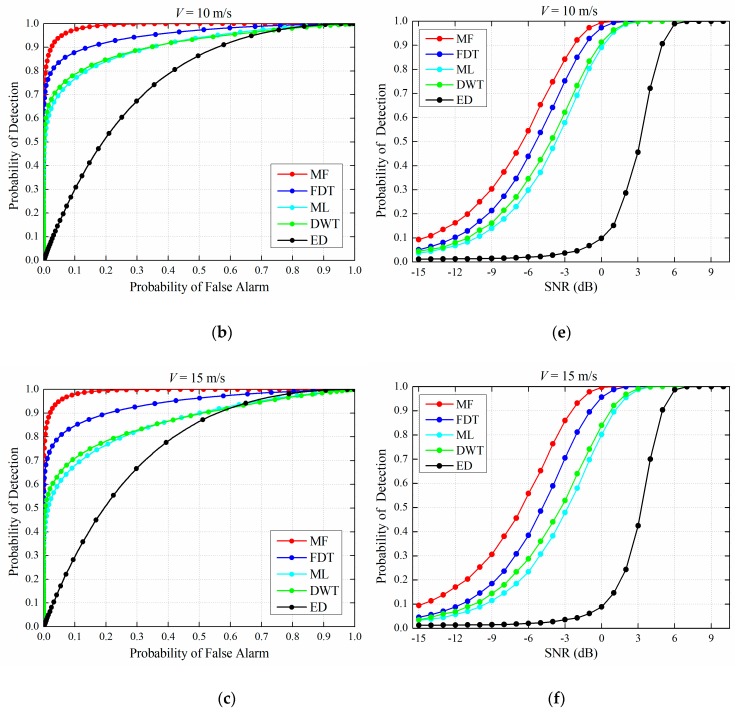
(**a**–**c**) Probability of detection (POD) versus probability of false alarm (PFA) for 5 m/s, 10 m/s and 15 m/s when signal-to-noise ratio (SNR) is ‒3 dB, respectively; (**d**–**f**) POD versus SNR in 5 m/s, 10 m/s and 15 m/s when PFA is 0.01, respectively.

The simulation parameters are chosen as follows: *N_x_* = 1200, *N_y_* = 2000, *β_max_* = 50. The FDT method is compared with other detection methods using the receiver operating characteristics (ROC) in the cases of 5 m/s, 10 m/s and 15 m/s, as shown in Figure 7a–c, respectively. Meanwhile, when the PFA is set to 0.01, the result of POD versus SNR is shown in Figure 7d–f. All results are obtained using Morte Carlo simulations with number of trial equals to 10^5^ times. According to the given SNR, the simulated narrow-band background noise is generated numerically for each trial. The range of SNR of interest here is from −3 dB to 0 dB. An SNR equal to 0 dB means that the signal and the noise have the same energy, and the SNR equal to −3 dB means that the signal energy is half of the noise energy. Thus, the performance evaluation is based on comparing the detection methods’ ability to accurately extract and identify the target ELF signals that are buried in the strong narrow-band background noise, which is critical for the practical AGM system, because it also requires the same ability to detect the short transient very weak tracking signals with very poor SNR.

Figure 7a–f shows that the POD of the FDT method is higher than the PODs of the ML and DWT methods, especially when the speed is up to 15 m/s. Compared with the ML and DWT methods, the POD of the FDT method exhibits a very small decrease as the speed of the transmitter increases at a given SNR or PFA. Figure 7a–f shows that the detection performance of the FDT method is close to the MF method (upper bound), which is very attractive. Taking Figure 7c as an example, when PFA is close to 0, the PODs of the MF and FDT methods are both high, corresponding to the upper left corner of Figure 7c. Taking Figure 7f as another example, when the SNR is 0 dB, they are both more than 95% in POD and the difference between them is less than 5%. An in-depth discussion on the main reasons for the obtained results will be presented in Section 5.

### 4.3. Field Testing and Validation

**Figure 8:**
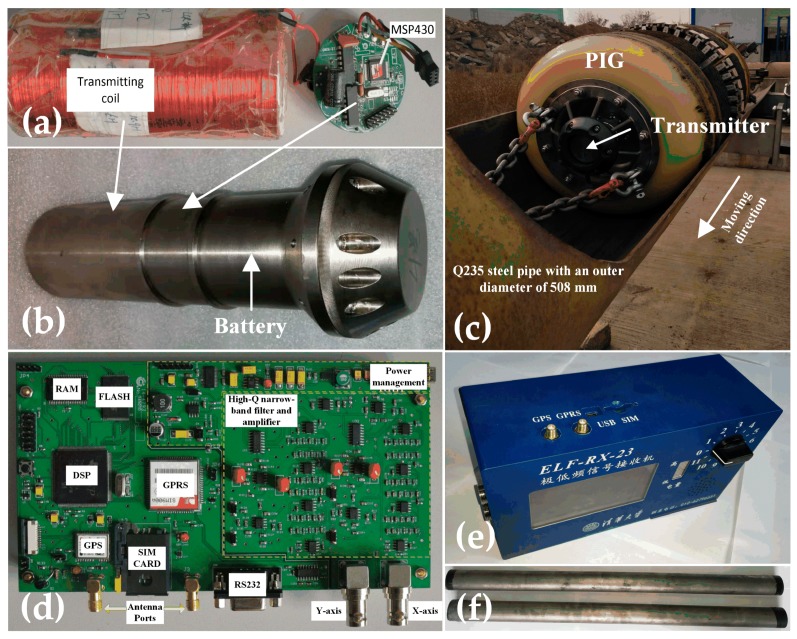
The newly designed and developed tracking system and 20˝ pipeline inspection gauge (PIG): (**a**) Transmitting coil and circuit; (**b**) ELF transmitter; (**c**) 20˝ PIG with transmitter; (**d**) Circuit of receiver; (**e**) ELF receiver; (**f**) A pair of search coil sensors.

The field testing is conducted by the newly designed and developed tracking system and 20˝ PIG by the authors’ group at Tsinghua University, as shown in Figure 8. The tracking system developed mainly includes an ELF transmitter, an ELF receiver and a pair of orthogonal search coil sensors. The transmitter generates the transmitting current which is a 23 Hz triangle wave using the H-bridge inverter controlled by MSP430 low power processor [43]. The titanium alloy shell is selected to protect the transmitter circuit for the high-pressure in-pipe situations due to the non-magnetic and lightweight properties. Figure 8c shows that the transmitter is equipped in the front of the PIG, and the PIG is pulled into the Q235 steel pipe at different speeds. Figure 8d,e shows that the ELF receiver mainly contains TMS320F28335 digital signal processor (DSP) and high-Q high-gain narrow-band filter and amplifier. The Q-factor of the designed 16th order band-pass filter is 16 (*f*_0_ = 23 Hz), and the overall gain of the multi-stage amplifier is 100,000 (100 dB). The *X*-axis and *Y*-axis ELF signals received from the orthogonal search coil sensors are first filtered and amplified, and then converted to digital signals by 12-bit A/D converter. The DSP chips are programed by the proposed FDT methods in C programming language. The digital ELF signals of the *X*-axis and *Y*-axis, and the test statistics, are recorded in real time. Figure 8f shows the developed search coil sensors, which also uses the titanium alloy shell for the protection.

**Figure 9:**
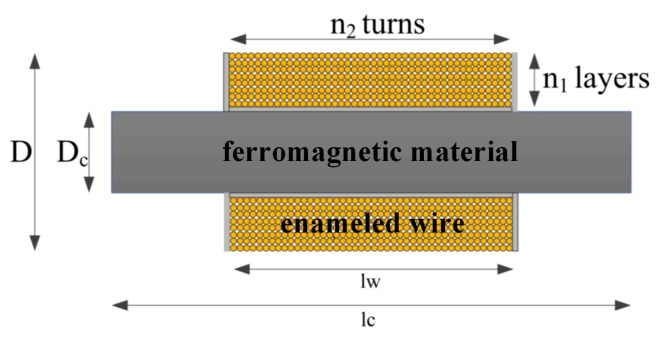
Structure of search coil sensor.

The narrow-long search coil sensor is developed to receive very weak ELF magnetic signals. The sensor is inserted by a rod of ferromagnetic material with a high magnetic permeability. The diameter of enameled wires is 0.25 mm. The structure is shown in Figure 9 and the parameters are listed in Table 5.

**Figure 10:**
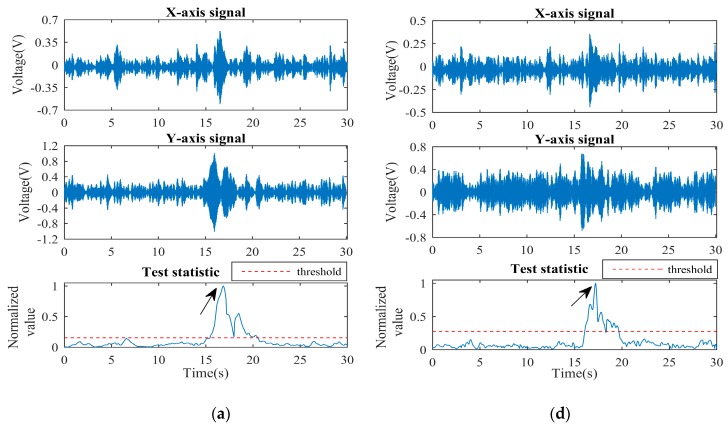

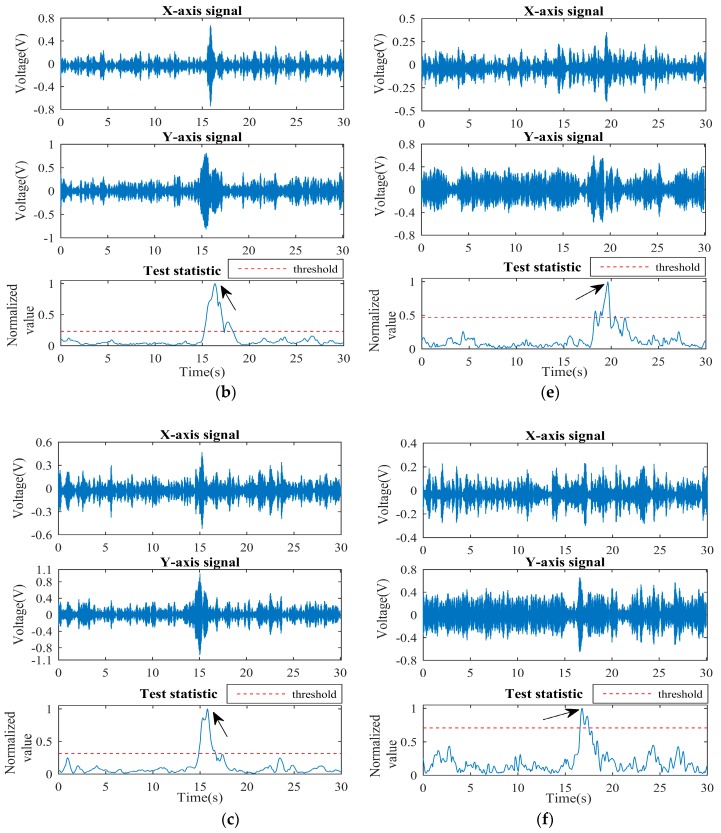
Time-domain ELF signals and normalized test statistics when PFA = 10^−4^: (**a**–**c**) High SNR situations with low, medium and high speeds, respectively; (**d**–**f**) Low SNR situations with low, medium and high speeds, respectively.

There were two groups of experiments: a high SNR situation with thin wall thickness, and a low SNR situation with thick wall thickness. Each group consisted of low speed (2 ~ 5 m/s), medium speed (5 ~ 10 m/s) and high speed (10 ~ 15 m/s), and the burial depth was 5 m. The experimental results are shown in Figure 10. The experimental parameters in Figure 10a–f, including pipe wall thickness and true speed, and the corresponding estimated parameters calculated by the receiver based on the proposed FDT method, are shown in Table 6. The true speed is recorded by the PIG odometer while the estimated speed is obtained from ${\hat{\beta }}_{\mathrm{xy}}$ of Table 4.

It is worth noting that when the normalized test statistic is equal to 1 and over the threshold (indicated by arrows), it represents that the transmitter is passing right below the receiver, and the receiver successfully detects the ELF signals in real time. Figure 10a–c shows that the *X*-axis ELF signal appears as an obvious unimodal characteristic while *Y*-axis ELF signal appears an obvious bimodal characteristic, which has the same characteristics as the 2-D FEM simulation. As the speed of the transmitter increases, the ELF signal envelopes appeared to be short transient and the durations of the test statistics exceeding the threshold were also shortened. Figure 10d–f shows that the ELF signals were very weak and heavily interfered by narrow-band background noise. For the case of Figure 10f shown in Table 5, the estimated SNR is even less than 0 dB. However, the normalized test statistic appears to be an obvious peak over the threshold around t = 17 s, which determines the hypothesis of the signal existence. At the same time and same measuring point, the ELF magnetic signals were also measured by Mag-03 flux-gate high-sensitivity magnetometer and found to be smaller than 10 pT. This result confirms that the developed tracking system can effectively detect the very weak ELF magnetic signals. The experiment results validate the proposed FDT method and the tracking system developed for high- sensitivity real-time PIG tracking.

## 5. Discussions

**Figure 11:**
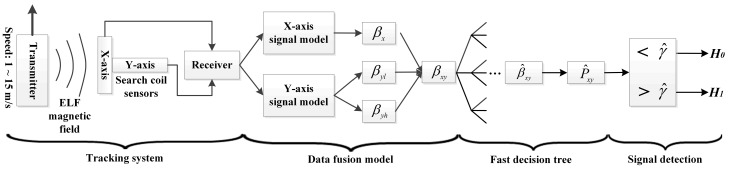
Flow chart of the proposed FDT method and tracking system developed.

The proposed FDT method and tracking system developed is summarized as the flow chart shown in Figure 11. The tracking system mainly developed relies on high-sensitivity search coil sensors, a high-Q narrow-band filter and high-gain amplifier, and the proposed FDT detection method to realize real-time detection of short transient very weak ELF magnetic signals. The 2-D FEM simulation results indicate that the magnitude of the very weak ELF magnetic signals is as low as 10 pT, which is lower than one millionth of the magnitude of the Earth’s magnetic field. Thus, the orthogonal search coil sensors, which have high number of turns, high-permeability iron core, and narrow long structure, are designed and developed to measure the very weak ELF magnetic signals along the *X*-axis and *Y*-axis. Then, the narrow-band filter and amplifier effectively eliminates out-of-band noise and amplify 23 Hz target signals. Finally, as shown in Figure 10d–f, even if the ELF signals are immersed in strong narrow-band background noise, the test statistics calculated from the FDT method are over the thresholds under a low PFA when the transmitter passes right below the receiver.

**Figure 12:**
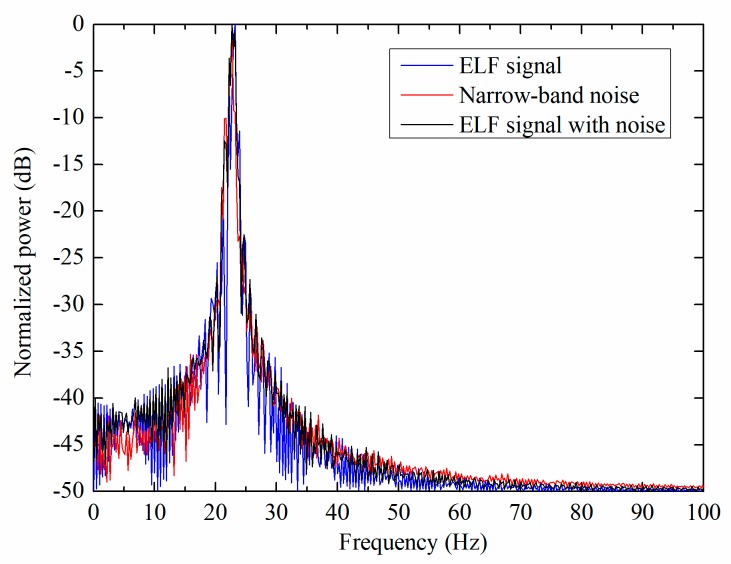
Normalized power spectrum when SNR is 0 dB and speed is 15 m/s.

The normalized power spectrum of the three different signals, including noiseless ELF signals, narrow-band noise and ELF signals with noise are shown in Figure 12. Since the frequency of narrow-band noise is concentrated at 23 Hz, it is difficult to discriminate between ELF signals and noise by frequency domain, which indicates that the frequency-domain information may not be useful for the detection. Thus, the performance of the time-frequency analysis based DWT method is lower than the time-domain characteristic based FDT method.

**Figure 13:**
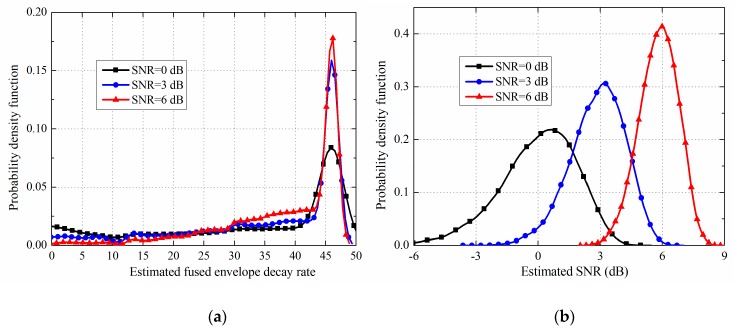
PDF of ${\hat{\beta }}_{\mathrm{xy}}$ and estimated SNR when the speed is 15 m/s: (**a**) ${\hat{\beta }}_{\mathrm{xy}}$; (**b**) Estimated SNR.

The probability density function (PDF) of ${\hat{\beta }}_{\mathrm{xy}}$ and estimated SNR is shown in Figure 13. Figure 13a shows a very concentrated distribution of ${\hat{\beta }}_{\mathrm{xy}}$ when the SNR is 6 dB or 3 dB, and even if the SNR is as low as 0 dB, the PDF also shows the concentrated distribution around 40~50 with a low variance (*β_xy_* is 46.9 from Table 4). The ${\hat{\beta }}_{\mathrm{xy}}$ is also used to estimate the real-time speed of the transmitter according to the relationship established between the 2-D FEM simulation results of ELF signals and the data fusion model. Then, Table 6 shows the errors between the estimated speed and the true speed are less than 1.5 m/s. Thus, these results are the main reason that the detection performance of the FDT method is higher than the amplitude-estimation based ML method and close to the upper theoretical bound. Meanwhile, it can be seen from Table 6 that the estimated SNR can effectively distinguish high SNR situations and low SNR situations, and can reflect the decrease of SNR as the speed increases because of the short transient characteristics. The reason for the good estimation performance of the SNR can also be verified by Figure 13b.

The real-time performance of the ELF tracking system developed is based on the data fusion model, fast calculation process, and the determined topology of the FDT. The data fusion model sets up the linear relationship between the *X*-axis signal model and the *Y*-axis signal model by fusing the envelope decay rates. Thus, the fused envelope decay rate can match well with the speed of the transmitter. Then, the fast calculation process was derived to calculate the *η* without massive matrix inversion and multiplication, and the calculation amount was found to be low enough for the DSP chip. The most important factor is that each tree nodes of the FDT is determined when the maximum speed of the transmitter is given. It is worth noting that the matrices including $H_{X}$ and $H_{Y}$, and the values including $P_{H_{X}}$ and $P_{H_{Y}}$ used for the calculation of the *η* and test statistic are both prior-known information because of the fast calculation process and determined FDT topology. Therefore, the proposed FDT method is very easily programmed, stored and calculated by the embedded DSP chip in practical applications. The experiments conducted by the receiver using a TMS320F28335 DSP chip finally validate the real-time performance of the FDT method.

## 6. Conclusions

Real-time detection of the short transient very weak ELF magnetic signals that are buried in strong narrow-band background noise is one of the key research areas in the nondestructive testing (NDT) field to ensure the safety of pipeline ILI for the oil and gas industry. The existing AGM systems work well for high-SNR and low-speed situations, but still face the challenges brought by high-sensitivity high-speed PIG tracking. This paper proposes a novel FDT detection method, as well as an ELF tracking framework for the high-speed PIG, to detect the short transient very weak ELF magnetic signals accurately and effectively. The 2-D FEM simulation results indicate that the duration and magnitude of the ELF signals can be as low as 1 s and 10 pT, which is validated by the experimental results. According to the short transient very weak characteristic as well as the linear relationship between *X*-axis and *Y*-axis ELF signal models, the data fusion model was established, and the fused envelope decay rate was used to match the speed of the transmitter. The proposed FDT method estimates the fused envelope decay rate to calculate the test statistic in real time, which was compared with the threshold to determine the existence of the ELF signals. The simulation results show that the FDT method has a higher POD than the other existing methods, especially when the speed is faster, for example, at 15 m/s. Meanwhile, the performance of the FDT method is close to the upper theoretical bound and the difference between them is less than 5% when SNR is 0 dB. The experimental results further validate that the proposed FDT method can detect the short transient very weak ELF signals in real time. Based on these results, the proposed FDT method and ELF tracking system developed are validated to be effective in real-time tracking of high-speed PIG.

## Figures and Tables

**Table 1:** Material parameters of 2-D FEM simulation model.

Property	Iron Core	Coil	Oil	Q235 Steel Pipe	Soil	Air
Conductivity (S/m)	1 × 10^7^	5.7 × 10^7^	0.01	2 × 10^6^	0.005	0
Permeability (H/m)	1000 *µ*_0_^1^	*µ*_0_	*µ*_0_	500 *µ*_0_	*µ*_0_	*µ*_0_
Mesh size (m)	0.01	0.01	0.1	0.02	0.2	0.2

^1^*µ*_0_*is permeability of vacuum*.

**Table 2:** Model geometry of 2-D FEM simulation model.

Property	Value
Length of pipeline (L)	45 m
Burial depth (D_B_)	2.5~5 m
Outer radius of pipeline (R)	254 mm
Thickness of pipe wall (T)	6~15 mm
Inner radius of pipeline	(R-T) mm
Length of coil (L_C_)	130 mm
Width of transmitting coil (2W_C_)	59 mm
Width of iron core (2W_I_)	25 mm
Coil turns	25,000
Transmitting current (RMS)	4 mA
Current frequency	23 Hz
Speed of transmitter (*V*)	1~15 m/s

**Table 3:** Notations used in this article.

Notations	Description
*N*_x_	Number of samples of *X*-axis ELF signal
*N_y_*	Number of samples of *Y*-axis ELF signal
*β_xy_*	Fused envelope decay rate
${\hat{\beta }}_{\mathrm{xy}}$	Estimated fused enveloped decay rate
*β_max_*	Fused envelope decay rate corresponding to maximum speed of transmitter
**H_x_**	Observation matrix of *X*-axis ELF signal
${\hat{P}}_{\mathrm{xy}}$	Maximized orthogonal signal power
$P_{H_{X}}$	Energy of *X*-axis observation vector
$P_{H_{Y}}$	Energy of *Y*-axis observation vector
*η*	Normalized signal energy
*C_m_*	Computation cost of multiplication
*C_a_*	Computation cost of addition
$\hat{\gamma }$	Threshold for signal detection

**Table 4:** Iteration procedure of the FDT method.

*L*		*V* = 5 m/s, *β_xy_* = 5.5			*V* = 10 m/s, *β_xy_* = 21.2			*V* = 15 m/s, *β_xy_* = 46.9		
1	${\hat{\beta }}_{\mathrm{xy}}$	**8.4**	25.2	42	8.4	**25.2**	42	8.4	25.2	**43**
	*η*	**0.9839**	0.8184	0.6975	0.9104	**0.9679**	0.9234	0.7881	0.9032	**0.9303**
2	${\hat{\beta }}_{\mathrm{xy}}$	2.8	**8.4**	14	**19.6**	25.2	30.8	36.4	42	**47.6**
	*η*	0.9703	**0.9839**	0.9288	**0.9704**	0.9679	0.9568	0.9261	0.9303	**0.9313**
3	${\hat{\beta }}_{\mathrm{xy}}$	**6.53**	8.4	10.27	17.73	19.6	**21.47**	45.73	**47.6**	49.47
	*η*	**0.9946**	0.9839	0.9676	0.9681	0.9704	**0.9709**	0.9312	**0.9313**	0.9310
4	${\hat{\beta }}_{\mathrm{xy}}$	**5.9**	6.53	7.16	20.84	**21.47**	22.09	46.98	**47.6**	48.2
	*η*	**0.9956**	0.9946	0.9920	0.9709	**0.9709**	0.9707	0.9513	**0.9513**	0.9512
Δ*η*		0.9956 − 0.9946 = 0.001			0.9709 − 0.9709 < 0.0001			0.9513 − 0.9513 < 0.0001		
Δ*β_xy_*^1^		5.9 − 5.5 = 0.40			21.47 − 21.2 = 0.27			47.6 − 46.9 = 0.70		

^1^ Δ*β_xy_ is error between β_xy_ (obtained from 2-D FEM simulation) and* ${\hat{\beta }}_{\mathrm{xy}}$ *(estimated from FDT method)*.

**Table 5:** Parameters of search coil sensor.

D (mm)	D_c_ (mm)	n_2_	n_1_	lw (mm)	lc (mm)
30	20	2400	23	720	800

**Table 6:** Estimated parameters of experimental results.

Figure 10	Pipe Wall Thickness	True Speed	Estimated Speed	Estimated SNR
(**a**)	10 mm	4.7 m/s	4.9 m/s	10.35 dB
(**b**)	10 mm	8.0 m/s	7.6 m/s	9.45 dB
(**c**)	10 mm	11.1 m/s	10.1 m/s	6.95 dB
(**d**)	15 mm	6.1 m/s	6 m/s	4.00 dB
(**e**)	15 mm	8.3 m/s	7.8 m/s	0.55 dB
(**f**)	15 mm	12.5 m/s	13.8 m/s	−0.9 dB
